# Leveraging observational data to identify targeted patient populations for future randomized trials

**DOI:** 10.21203/rs.3.rs-2641628/v1

**Published:** 2023-05-05

**Authors:** Daniel V. Lazzareschi, Nicholas Fong, Romain Pirracchio, Michael R. Mathis, Matthieu Legrand

**Affiliations:** UCSF: University of California San Francisco; UCSF: University of California San Francisco; UCSF: University of California San Francisco; U-M: University of Michigan; University of California San Francisco

**Keywords:** albumin, pragmatic approach to clinical trials, generalizability, randomized controlled trials

## Abstract

Randomized controlled trials reported in the literature are often affected by poor generalizability, and pragmatic trials have become an increasingly utilized workaround approach to overcome logistical limitations and explore routine interventions demonstrating equipoise in clinical practice. Intravenous albumin, for example, is commonly administered in the perioperative setting despite lacking supportive evidence. Given concerns for cost, safety, and efficacy, randomized trials are needed to explore the clinical equipoise of albumin therapy in this setting, and we therefore present an approach to identifying populations exposed to perioperative albumin to encourage clinical equipoise in patient selection and optimize study design for clinical trials.

## INTRODUCTION

Randomized controlled trials reported in the literature are often affected by poor generalizability, and pragmatic trials have become an increasingly utilized workaround approach to overcome logistical limitations and explore routine interventions demonstrating equipoise in clinical practice^1^. Intravenous albumin, for example, is commonly administered in the perioperative setting despite lacking supportive evidence. Given concerns for cost, safety, and efficacy, randomized trials are needed to explore the clinical equipoise of albumin therapy in this setting, and we therefore present an approach to identifying populations exposed to perioperative albumin to encourage clinical equipoise in patient selection and optimize study design for clinical trials.

## METHODS

We retrospectively analyzed 614,215 major non-cardiac surgeries performed under general anesthesia between January 2014 and June 2020 in 54 U.S. member hospitals of the Multicenter Perioperative Outcomes Group^2^. Major surgery was defined by anesthetic duration ≥ 2 hours and OPT base units ≥ 5. Patients with estimated glomerular filtration rate (eGFR) ≤ 15 mL/min/1.73m^2^, American Society of Anesthesiology (ASA) physical status 6, repeat surgery within 30 days, or cardiac, obstetric, neurosurgical, or liver transplant procedures were excluded (Supplemental Fig. 1). The study aims to identify and explore the interconnectedness of factors associated with the use of perioperative albumin.

We performed a mixed-effects logistic regression adjusting for institution as a random effect to explore patient, surgical, and anesthetic covariates associated with albumin use. These covariates included basic demographic information, surgical subspecialty service, preexisting patient comorbidities as phenotyped by the ICD-based Elixhauser index^3^, impaired renal clearance (eGFR < 60mL/kg/1.73mm^2^), anemia (hemoglobin < 12 g/dL), thrombocytopenia (platelets < 100×10^3^/mm^3^), emergent nature of surgery, complicated ASA physical status (≥ 4), large-volume crystalloid resuscitation (≥ 30mL/kg), significant blood loss (≥ 500mL), large-volume blood product transfusion (≥ 1L), the use of ≥ 1 continuous vasopressor infusions, and the cumulative dose of vasopressors expressed in norepinephrine equivalents. Imputation was not used for missing data, and observations with missing values involving albumin administration or acute kidney injury (AKI) were excluded from analysis.

## RESULTS

**Figure 1:**
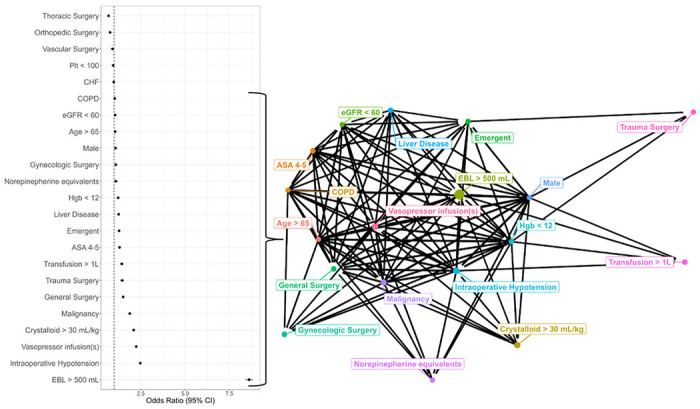
Forest plot and network diagram depicting factors associated with albumin use. The forest plot depicts adjusted odds ratios (aOR) of demographic, comorbid, surgical, and hemodynamic predictors of albumin administration with accompanying network diagram. The network diagram depicts binary variables with aOR >1.0 (all listed aORs on forest plot except for norepinephrine equivalents, which is continuous). Nodal size reflects the odds of receiving albumin attributable to that variable (aOR – 1.0) whereas edge thickness connecting nodes reflects the number of cases sharing those traits (n). **ABBREVIATIONS**: CKD = chronic kidney disease; CHF = congestive heart failure; eGFR = estimated glomerular filtration rate; EBL = estimated blood loss; ASA = American Society of Anesthesiologists classification; Hgb = hemoglobin (g/dL); Plt = platelets (x10^3^/mm^3^) DEFINITIONS: Norepinephrine equivalents (NEE): NEE = [norepinephrine (mcg/kg)] + [epinephrine (mcg/kg)] + [dopamine (mcg/kg)]/150 + [phenylephrine (mcg/kg)]/10 + [vasopressin (U)]/[0.4*weight (kg)] Intraoperative hypotension: Defined as one or more episodes of mean arterial pressure less than 65 mmHg for a duration at least 15 minutes.

Intraoperative albumin was used in 15.3% of all procedures. Factors associated with albumin use included patient age ≥ 65 years (aOR 1.05, 95% CI 1.03–1.07), ASA status ≥ 4 (1.3, 1.26–1.35), male sex (1.08, 1.06–1.10), impaired renal function (1.05, 1.02–1.08), chronic obstructive pulmonary disease (1.03, 1.00-1.05), liver disease (1.25, 1.21−1.29), malignancy (1.88, 1.84–1.92), anemia (1.22, 1.20–1.24), emergent surgery (1.27, 1.23–1.32), large-volume crystalloid (2.1, 2.02–2.18), intraoperative hypotension (2.47, 2.42–2.52), large-volume blood loss (8.61, 8.40–8.82), large-volume blood product transfusion (1.43, 1.33–1.53), cumulative vasopressors (1.09, 1.06–1.12), the use of ≥ 1 vasopressor infusions (2.24, 2.19–2.30), and finally, general (1.51, 1.47–1.55), gynecologic (1.09, 1.05–1.13), and trauma (1.45, 1.47–1.55) surgical subspecialties (Supplemental Table 1). We created a network diagram demonstrating the interconnectedness of factors associated with albumin use. Hemodynamic variables – hypotension, vasopressors, crystalloid volume, and blood loss – demonstrated the greatest degree of interconnectedness, while baseline and preoperative patient characteristics were peripherally connected and appeared less likely to prompt albumin use (Fig. 1). The maximum rate of missingness among included covariates was 6.2%.

## DISCUSSION

The use of albumin colloids remains largely unexplored in the perioperative setting. Pharmacokinetics describe a rapid and prolonged duration of plasma expansion by albumin compared to crystalloids^4–7^, yet studies exploring albumin use in ICU populations have not revealed significant differences in overall fluid balance^8^, and starch-based colloids are associated with increased risk of bleeding, AKI, and death^9^.

Even when viewed through the lens of rigorous causal inference techniques, associations described in observational studies suffer from residual confounding and therefore merit cautious interpretation^10^. Given the nontrivial cost of albumin therapy, its routine use in medical centers, and the conspicuous lack of evidence standardizing its clinical use, randomized trials are urgently needed to explore the impact of intraoperative albumin on postoperative outcomes. A large pragmatic trial would ideally target the population of interest for which equipoise exists, and our findings could help design such a trial. Importantly, while several baseline characteristics were associated with albumin use, intraoperative factors – including large-volume crystalloid resuscitation, continuous vasopressor infusions, and procedures involving significant blood loss – appear to play a key role in the decision to administer albumin and therefore describe a population in which equipoise is likely to exist for this therapy. Selecting patients based on preoperative characteristics would therefore fail to maximize equipoise in the enrolled population, and trial design should instead account for relevant intraoperative factors to ensure results are able to inform clinical practice.

## CONCLUSIONS

While we presently describe an empirically defined target population tailored to perioperative albumin exposure with appropriate equipoise for trial design, similar methods could be extended to explore other routine perioperative interventions of unclear safety and efficacy that warrant further investigation.

## Data Availability

The datasets used and/or analyzed during this study are available from the corresponding author on reasonable request.

## Abbreviations

AKI: Acute Kidney Injury
ASA: American Society of Anesthesiology
CPT: Current Procedural Terminology
eGFR: estimated Glomerular Filtration Rate
MPOG: Multicenter Perioperative Outcomes Group
